# 

**Published:** 1871-03

**Authors:** 


CONTENTS:

ORIGINAL CONTRIBUTIONS.

A Brief Paper on the Comparative Effects of Alcohol and Tobacco, on the Human System. By N. S. Davis, M.D., Chicago, Ill. .......... 129
Climate of Nebraska—Cerebral and Bowel Affections, etc. By Noble Holton, M.D., Falls City, Neb. .......... 142
Sulphate Morphia as a Parturient. By L. D. Robinson, M.D., Coatsville Ind. 148
Rupture of the Uterus—Gastrotomy, .... 149
Comments of "W. H.," Assistant-Editor of the Chicago Medical Journal, Feb., 1871, upon Opium in Metro-Peritonitis, Briefly Reviewed. By Theodore Griffin, M.D., Chicago, Ill. .......... 150
Ossification of the Choroid; Eneucleation of the Globe. By C. Hixson, M.D., Kansas City, Mo., .......... 155

THE CLINIC.

Clinical Cases in Medical Wards of Mercy Hospital. By Prof. N. S. Davis:
Case I—Chronic Army Diarrhœa, ...... 156
Case II—Chronic Rheumatism, ........ 158
Case III—Anemia and Anasarca, ..... 160

CORRESPONDENCE.

Letter from F. K. Bailey, M.D., ........ 162
Letter from D. B. Trimble, M.D., ....... 166

SELECTIONS.

Cæsarean Operation Performed four times in the same Subject, .. ......... 141
Influence of Diet on the Composition of Bone. .......... 168
Hemiphlegia from Cerebral Hemorrhage in Cases where there is Valvular Disease of the Heart .......... 169
Epidemic of Chorea Minor, .......... 169
Pathology of Angina Pectoris, .......... 170
Influence of Digitalis on Nutrition, ..... 170
Chloral in Midwifery, .......... 171
Rapid Cure of Buboes, .......... 171
Treatment of Inflamed Testicle by Puncture, .......... 171

ABRIDGMENTS FROM EXCHANGES.

Recovery from a Scalp Wound, ......... 172
Permanganate of Potassa, .......... 172
Pen Pictures of Classic Men and Classic Scenes in Europe, .......... 173
Artificial Fecundation, .......... 180

BOOK NOTICES.

Spermatorrhœa: Its Causes, Symptoms, Results, and Treatment. By Roberts Bartholow, A.M., M.D., .......... 181
On Diseases of the Spine and of the Nerves. By Charles B. Radcliffe, M.D., F.R.C.P., .......... 181
First Medical and Surgical Report of the Boston City Hospital; Edited by J. Nelson Borland, M.D., and David W. Cheever, M.D., .......... 181

EDITORIAL.

Medical Society of District of Columbia, 182
Errors, .......... 183
Good Appointment, .......... 183
Chloroform Deaths, .......... 183
College Commencement Exercises, ..... 184
Chicago Woman's Hospital Medical College, .......... 184
Chicago Medical College—Medical Department of N. W. University, . ...... 184
Meeting of Alumni Association of Chicago Medical College, .......... 185
Medical Association of East Tennessee, 185
American Medical Association, .......... 185
Obituary, .......... 187
Sex of the Fœtus in Uteri Determined by the Number of Cardiac Pulsations, .... 188
Mortality for the Month of January, 1871, 189
Money Receipts, .......... 190

